# Exercise Influences the Brain’s Metabolic Response to Chronic Cocaine Exposure in Male Rats

**DOI:** 10.3390/jpm14050500

**Published:** 2024-05-09

**Authors:** Aidan Powell, Colin Hanna, Munawwar Sajjad, Rutao Yao, Kenneth Blum, Mark S. Gold, Teresa Quattrin, Panayotis K. Thanos

**Affiliations:** 1 Behavioral Neuropharmacology and Neuroimaging Laboratory on Addictions, Department of Pharmacology and Toxicology, Clinical Research Institute on Addictions, Jacobs School of Medicine and Biomedical Science, State University of New York at Buffalo, Buffalo, NY 14203, USA; apowell7@buffalo.edu (A.P.); cshanna2@buffalo.edu (C.H.); 2 Department of Nuclear Medicine, University at Buffalo, Buffalo, NY 14214, USA; msajjad@buffalo.edu (M.S.); rutaoyao@buffalo.edu (R.Y.); 3 Center for Sports, Exercise, and Mental Health, Western University of Health Sciences, Pomona, CA 91766, USA; drd2gene@gmail.com; 4 Department of Molecular Biology, Adelson School of Medicine, Ariel University, Ariel 40700, Israel; 5 Department of Psychiatry, Washington University School of Medicine, St. Louis, MO 63110, USA; drmarksgold@gmail.com; 6 UBMD Pediatrics, JR Oishei Children’s Hospital, University at Buffalo, Buffalo, NY 14203, USA; tquattrin@upa.chob.edu

**Keywords:** 18F-FDG fluorodeoxyglucose, positron emission tomography, aerobic exercise, glucose metabolism, statistical parametric mapping, cocaine

## Abstract

Cocaine use is associated with negative health outcomes: cocaine use disorders, speedballing, and overdose deaths. Currently, treatments for cocaine use disorders and overdose are non-existent when compared to opioid use disorders, and current standard cocaine use disorder treatments have high dropout and recidivism rates. Physical exercise has been shown to attenuate addiction behavior as well as modulate brain activity. This study examined the differential effects of chronic cocaine use between exercised and sedentary rats. The effects of exercise on brain glucose metabolism (BGluM) following chronic cocaine exposure were assessed using Positron Emission Tomography (PET) and [18F]-Fluorodeoxyglucose (FDG). Compared to sedentary animals, exercise decreased metabolism in the SIBF primary somatosensory cortex. Activation occurred in the amygdalopiriform and piriform cortex, trigeminothalamic tract, rhinal and perirhinal cortex, and visual cortex. BGluM changes may help ameliorate various aspects of cocaine abuse and reinstatement. Further investigation is needed into the underlying neuronal circuits involved in BGluM changes and their association with addiction behaviors.

## 1. Introduction

Cocaine use is associated with a wide range of negative health outcomes [1]. The number of deaths involving cocaine has also increased steadily since 2015, with 24,486 deaths reported in 2021. Currently, there is no approved treatment for cocaine overdose. In the United States, there are an estimated 2 million regular users of cocaine, with no approved medication for the treatment of cocaine use disorder (CUD) [2]. Cocaine is often used with alcohol, opioids, and benzodiazepines, making overdose and treatment failures more likely. Psychosocial treatment of CUD has high dropout and recidivism rates, and no pharmacological treatments have been approved [2]. Researchers are currently investigating both pharmacological and non-pharmacological approaches to treating addictions and substance use disorders such as CUD [2,3,4,5].

Previous studies have shown that aerobic exercise alters BGluM in regions associated with sensory processing, motor function, and motivated behavior, both alone [6] and in response to acute cocaine [7].

In female Lewis rats, exercise has been shown to increase brain glucose metabolism in a range of sensorimotor regions [6,7,8]. In rats chronically exposed to cocaine, exercised rats showed activation in the secondary visual cortex, lateral area (V2L) compared to their sedentary counterparts [8,9]. Our group previously found the modulation of BGluM in sensory cortical areas following both exercise alone and acute cocaine [6,7]. Other sensorimotor regions, such as the central nucleus of the inferior colliculus (CIC), caudate putamen (striatum) (CPu), and primary auditory cortex (Au1), have been found to be activated in response to chronic aerobic exercise alone [6]. In chronically exercised female rats exposed to an acute dose of cocaine, activation has been observed in the temporal association area (TeA), entopeduncular nucleus (EP), Crus 1 of the ansiform lobule (crus 1), and substantia nigra. The visuospatial demand required for exercise may explain the activation of sensorimotor regions and coincides with the acute effects of exercise proposed by the “transient hypofrontality hypothesis” [10]. This hypothesis proposes that metabolic resources are redirected from regions not pertinent to exercise performance to structures that are required for motor patterns, the assimilation of sensory inputs, and the coordination of autonomic regulation. Increased BGluM in the sensorimotor regions has also been observed in works from the literature investigating the effects of physical exercise on humans, indicating potential clinical relevance [11]. In contrast, various sensorimotor regions have been observed to be inhibited in female rats exposed to both chronic exercise and chronic cocaine. BgluM inhibition has been observed in relation to the paraflocculus (PFL), the eighth cerebellar lobule (8cb), the paramedian lobule [2], the copula of the pyramis (COP), the stria terminalis (st), the stria medullaris of the thalamus, the medial and posteromedial parts of the bed nucleus of the stria terminalis (stmpm), the ventrolateral thalamic nucleus, (VL), the primary somatosensory cortex, hindlimb region (S1HL) [8]. 

The literature suggests that glucose metabolism differs based on the history of cocaine use. This has been demonstrated in a study on BgluM changes in Rhesus monkeys after the administration of an acute cocaine injection, 60 sessions of self-administration under limited-access conditions (1 h/day), 60 sessions under extended-access conditions (4 h/day), and 4 weeks of abstinence [12]. BgluM inhibition was observed in the prefrontal cortex, expanding to other regions of the frontal cortex with higher levels of cocaine consumption. In humans, cocaine abuse is associated with decreased glucose metabolism in the frontal cortex, which persists for 3–4 months after abstinence [13]. The frontal lobe itself is acutely affected by cocaine, and the effects persist the longest in the region during withdrawal. As previously mentioned, a range of sensorimotor regions have been shown to be inhibited in female rats exposed to chronic cocaine and chronic exercise [8]. The inhibition of regions outside of the frontal lobe could help correct the biased inhibition of the frontal lobe observed In humans and non-human primates following chronic cocaine use, particularly if regions are functionally connected to the frontal lobe. Furthermore, regions associated with motivated behavior have been shown to be modulated in our group’s previous findings [8]. Inhibition in cPu, st, and the thalamus are of particular interest because they are a part of the brain’s reward cascade [14], which may impact response to cocaine. These results in female rats imply that exercise could help balance the bGluM changes observed following chronic cocaine [15] or psychostimulant use [16,17]. The functional connectivity of regions affected, as well as the role of specific regions in addiction, should also be considered [9].

Based on the previous literature, changes in basal ganglia activity associated with cocaine would be expected to be attenuated. The activation of the caudate putamen (striatum) (CPu) in chronically exercised female Lewis rats compared to sedentary controls [8] is of particular interest because the region is a part of the basal ganglia, which has been implicated in various aspects of cocaine-induced behavioral [18] effects. In female rats administered chronic cocaine in conjunction with the same exercise regimen as used in the current study, various regions of the basal ganglia were inhibited. Due to the role of the basal ganglia in reward-seeking [19], the modulation of BGluM in this region is of interest in terms of determining the efficacy of exercise in attenuating addiction behavior. While previous work by our group demonstrated results supporting these mechanisms of action in female rats, male rats remain to be investigated, which is the purpose of the current study. Due to sex differences in the effects of cocaine and susceptibility to abuse [20,21,22], sex-specific studies are required to assess the use of exercise as an intervention [23].

Behavioral results support the hypothesis that exercise has a beneficial role in drug addiction, as exercise has been shown to decrease drug maintenance and the acquisition of cocaine self-administration [24] in adolescent and adult rats [25]. Binge use of alcohol in adult rats has also been shown to be reduced by exercise [26]. Alcohol consumption in a two-bottle choice model has been shown to be reduced in mice given access to running wheels [27]. Maintenance of methamphetamine self-administration in rats is attenuated when given access to running wheels; however, this is only the case if access is available at the start of self-administration [28]. 

The impact of exercise on addiction behavior has been shown to depend on the phase of the addiction process when used as an intervention [29]. The protective effects of exercise during initiation and withdrawal are attributable to its effect on dopaminergic transmission. Physical exercise has been shown to attenuate withdrawal symptoms as well as increase abstinence rates when used as an intervention in various substance use disorders [30]. In rats, exercise has been shown to reduce cocaine place preference, cue-induced reinstatement, and locomotor responses [4,5]. Aerobic exercise has been shown to reduce rates of cocaine acquisition and have a protective effect on cocaine-seeking at both low and high doses of self-administration [25]. 

At a neuronal signaling level, exercise has been shown to have a range of beneficial effects on drug-induced changes in neurotransmission. During drug use, exercise has been shown to help normalize glutamatergic and dopaminergic transmission in the reward pathway and reverse the modification of chromatin [31,32,33,34]. Aerobic exercise has been shown to produce rewarding effects by activating glutamate neurons in the red nucleus, which project to dopaminergic neurons in the VTA [35]. Chronic drug exposure is also associated with glutamatergic over-stimulation of the reward system, which may be corrected by exercise and has been shown to decrease striatal and hippocampal glutamate concentrations [35,36]. Exercise produces non-drug reward-producing changes in the mesolimbic reward pathway, which is associated with altered sensitivity to drugs of abuse [37]. However, the effect of exercise on the reward system is not completely beneficial, as exercise-induced plasticity has been shown to intensify drug associations if drug exposure occurs after a chronic exercise regimen. For example, a history of chronic voluntary exposure to wheel running has been shown to enhance conditioned place preference for morphine and cocaine in rats [38,39]. Chronic drug exposure is associated with reduced dopamine release, which may promote further drug use and withdrawal symptoms to correct for a lack of dopaminergic signaling [40,41]. Cocaine dependence, in particular, is associated with dopamine depletion [42,43]. The increased dopaminergic signaling caused by exercise may help correct the dysfunction seen in chronic drug exposure and reduce withdrawal symptoms. The potentiation of dopaminergic signaling has been shown to attenuate alcohol self-administration [44,45], and exercise may help in a similar manner. 

Understanding the underlying mechanisms of the effects of exercise on cocaine-related behavior can be facilitated by gaining more insight into how exercise modulates various circuits in the brain. Positron Emission Tomography (PET) has the ability to assess how exercise modulates specific brain regions, assisting our understanding of the underlying mechanisms that dictate how exercise affects cocaine and addiction-related behavior. [18F]-Fluorodeoxyglucose (FDG) was used in the current study to assist in observing the changes in brain glucose metabolism (BGluM). This experiment served to advance our understanding of the underlying mechanisms by which aerobic exercise impacts subsequent cocaine exposure, identifying potential targets for further anatomical investigation in the ongoing research looking at exercise for addiction treatment. The data suggest that chronic aerobic exercise helps attenuate the effects of hypoactivation in the frontal cortex in addition to impacting regions involved in addiction behavior. This study aims to identify if similar regions are involved in the exercise-mediated inhibition of addiction behavior observed in male rats using an identical protocol that has been previously used for females.

## 2. Materials and Methods

### 2.1. Animals

Eight-week-old young adult male rats (N = 16) were obtained from Taconic (Hudson, NY, USA). The animals were split into an exercised group (N = 8) and a sedentary group (N = 8). The animals were individually housed at room temperature ~22 °C on a 12 h reverse light/dark cycle. The dark cycle was from 6 a.m. to 6 p.m. The rats were given unlimited access to food and water in their home cages and were handled daily. The animals weighed approximately 250–285 g at the start of the study. They were allowed one week to habituate to their environment before beginning the assigned group regimens. This experiment was conducted in accordance with the National Academy of Sciences Guide for the Care and Use of Laboratory Animals (1996) and approved by the University at Buffalo Institutional Animal Care and Use Committee (Approval code: RIA 13095Y; Approval date: 1 July 2023).

### 2.2. Exercise

Exercise was the experimental variable. The animals in the exercised group (N = 8) underwent the following regimen: Forced running was performed on a customized treadmill divided into individual plexiglass running lanes. The exercise regimen started at 10 min a day at 10 m/min, increasing by 10 min each day until a maximum time of 1 h was reached, as per the standard exercise protocol [6,8]. The animals were given a ten-minute break after 30 min of running. This exercise regimen was maintained for 5 days per week for six weeks [8]. Sedentary rats remained in their home cages for the duration of the exercise regimen, receiving no treadmill exercise, as previously described [4,6,46,47].

### 2.3. Chronic Cocaine Treatment

Prior to imaging, all of the rats underwent chronic cocaine exposure. The cocaine was dissolved in 0.9% saline and injected via the intraperitoneal route at 25 mg/kg, consistent with previous experiments administering chronic cocaine over similar time periods [48], and with publications using the current exercise protocol [8]. Cocaine administration occurred daily for 8 days, alternating every other day with saline. Four total cocaine injections were administered.

### 2.4. PET Imaging

The rats were food-restricted for 8 h prior to imaging to normalize blood glucose levels. The rats were then given 500 ± 115 μCi of 18F-FDG via intraperitoneal injection. A 30 min uptake period followed the injections, and the animals were anesthetized immediately after. The rats were anesthetized with 3% isoflurane, maintained at 1% throughout the scan. PET imaging was completed using a microPET^®^ Focus120 scanner (Concorde microSystems Inc. (Knoxville, TN, USA), transaxial resolution: 1.3 mm full-width at half maximum, transaxial field view: 8.0 cm). Anesthetized rats were secured to the scanner bed for 30 min, as per the standard imaging protocol.

### 2.5. PET Image Analysis

**Figure 1:**
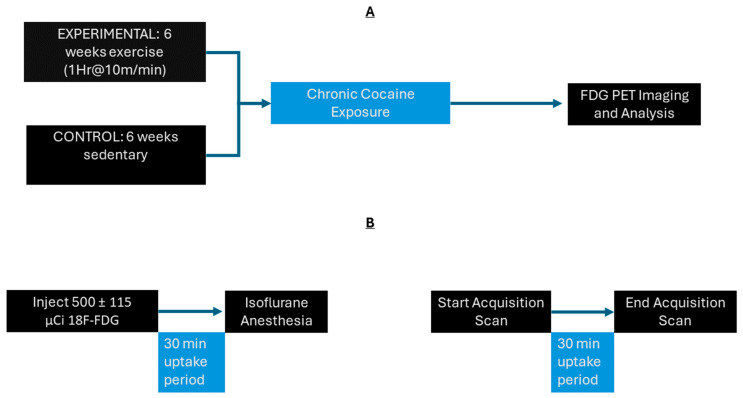
Experimental timeline: (**A**) The rats were split into exercise and sedentary groups. Exercise group rats underwent 6 weeks of exercise, and sedentary rats remained in their home cages. All animals underwent chronic cocaine exposure for 8 days followed by microPET scans. (**B**) Timeline of PET scans: rats were given [18F]-Fluorodeoxyglucose (FDG). Then, a 30 min uptake period followed. Animals were then anesthetized with isoflurane (3%), maintained throughout the duration of the 30 min PET scan (1%). This was based on a standard protocol, as described in [8].

Analysis was conducted as previously described [6,49,50]. The scans were reconstructed using the MAP algorithm (15 iterations, 0.01 smoothing, 256 × 256 × 256 resolution). The reconstructed scans were manually coregistered onto a rat brain MRI template (63 slices, Paxinos and Watson Stereotaxic coordinates) in PMOD imaging software. (Version 2.85, PMOD technologies, Fällanden, Switzerland). Automatic coregistration and spatial normalization were carried out using MATLAB software (MATLAB, R2018b). Statistical Parametric Mapping (Voxel Threshold, K > 50) in MATLAB was then used to find significant differences in cluster size between the exercise and sedentary groups. Using PMOD software, significant clusters were again fitted onto the rat brain MRI template. Clusters were then mapped and labeled using “The Rat Brain in Stereotaxic Coordinates” atlas [51]. A complete experimental timeline can be viewed in Figure 1.

## 3. Results

### Statistics

**Figure 2:**
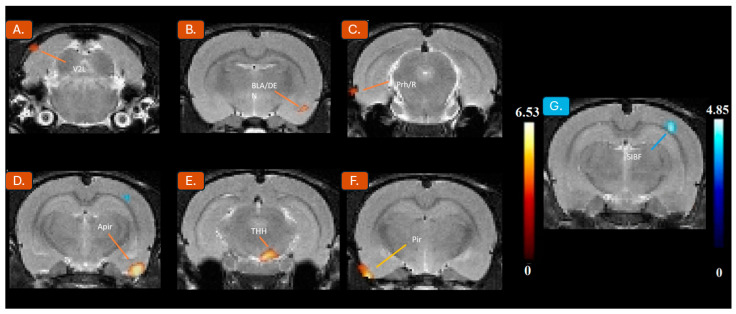
Significant activation clusters: coronal PET images showing brain regions with significant (*p* < 0.001, K > 50) metabolic increases (**A**–**F**) in exercised rats compared to sedentary rats labeled in orange. T-values represent peak activation (t = 6.53). Hot scale clusters illustrate BGluM activation in the (**A**) V2L, (**B**) BLA/DEN, (**C**) Prh/R, (**D**) Apir, (**E**) THH, and (**F**) Pir. Significant inhibition clusters: coronal PET images showing brain region with significant (*p* < 0.001, K > 50) metabolic decreases (**G**) in exercised rats compared to sedentary rats labeled in blue. T-values represent peak inhibition (t = 4.85). Cold-scale clusters illustrate BGluM reduction in (**G**) SIBF.

A two-sample t-test revealed that the exercised rats showed significantly increased BGluM (*p* < 0.001, K > 50) compared to sedentary rats in the following regions (See Figure 2): amygdalopiriform transition area (t = 6.39; z = 3.83; KE = 244), basolateral amygdaloid nucleus/dorsal endopiriform nucleus piriform cortex layer 1 (t = 6.53; z = 3.87; KE = 94), trigeminothalamic tract (t = 5.57; z = 3.58; KE = 178), perirhinal cortex/rhinal fissure (t = 4.58; z = 3.21; KE = 112), and secondary visual cortex, lateral area (t = 4.17; z = 3.03; KE = 50). The only region with a significant decrease (See Figure 2) in BGluM was the SIBF primary somatosensory cortex (t = 4.85; z = 3.32; KE = 99). Information regarding cluster location, size, and statistical significance is reported in Table 1.

## 4. Discussion

The results of the FDG PET analysis of male rats found differential activation in the exercised group compared to the sedentary group under chronic cocaine treatment. While sample size may be considered a limitation, it is consistent with previous publications [8]. Housing and PET imaging protocols were identical between the groups to minimize methodological bias. The animals were also handled daily to minimize stress. Regions associated with drug cue-induced reinstatement, drug cue-induced conditioning, and compulsive drug use appear to be normalized. Additionally, a handful of affected regions border and project into the frontal lobe, which may imply the prevention of the more acute effects of cocaine. The SIBF primary somatosensory cortex (SIBF) was found to be inhibited in the exercised group. Increased activation was observed in the amygdalopiriform transition area (Apir), piriform cortex layer 1, trigeminothalamic tract (TTH), perirhinal cortex rhinal fissure (Prh/Rf), basolateral amygdaloid nucleus dorsal endopiriform nucleus (BLA/DEN), and secondary visual cortex, lateral area (V2L). Our group conducted the same protocol with female rats and found the exercised group to only have significant activation in the V2L compared to sedentary counterparts. Inhibition occurred in the paraflocculus (PFL), the eighth cerebellar lobule (8cb), the paramedian lobule [2], the copula of the pyramis (COP), the stria terminalis (st), the stria medullaris of the thalamus, the medial and posteromedial parts of the bed nucleus of the stria terminalis (stmpm), the ventrolateral thalamic nucleus, (VL), and the primary somatosensory cortex, hindlimb region (S1HL) [8].

In female cohorts subjected to the same protocol [8], sensorimotor regions were more activated in the exercised group compared to the sedentary group following chronic cocaine exposure. In contrast, in the male rats of the current study, sensory regions such as the V2L and Pir were found to be activated. These are associated with visual [52] and olfactory [53] sensory processing, respectively. Regions associated with higher-order sensory processing were also found to be activated. Prh/Rf, which is involved in object recognition memory [54], was found to have increased BGluM. Apir, which is associated with the processing of olfactory and gustatory information [55], was activated. The BLA/DEN is associated with integrating sensory stimuli and emotional responses [56]. TTH was found to be more activated in the exercised group, and it provides motor innervation to the jaw and is involved in orofacial nociception [57,58].

**Figure 3:**
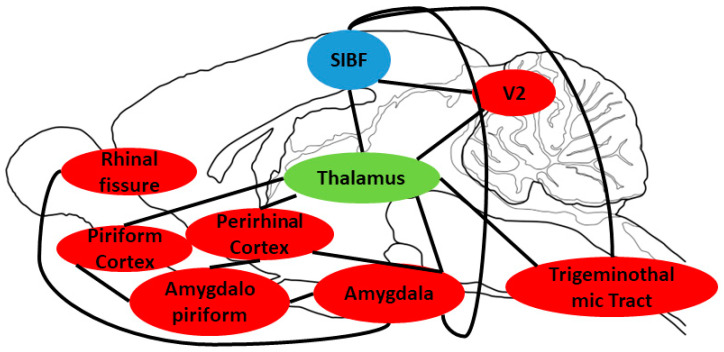
Hypothesized brain circuitry following chronic cocaine and exercise on a sagittal drawing. Activated/increased BGluM clusters are shown in red, while the inhibition of BGluM is shown in blue. Green boxes are brain regions that may serve as connection points between clusters.

An interesting similarity between sexes is the inhibition of the somatosensory cortex. In the somatosensory cortex, acute cocaine is known to decrease spontaneous background neuronal activity [59]. Exercise appears to enhance the ability of the somatosensory cortex to ignore background stimulus [60], which may exacerbate the above-mentioned effects of cocaine. However, it is unclear if the observed exacerbation of inhibition will result in adverse effects on cocaine addiction as connectivity between other regions is also a factor associated with addiction behavior. Chronic cocaine self-administration is associated with hypoconnectivity between the somatosensory cortex and dorsal anterior cingulate, with higher consumption correlating to increased hypoconnectivity [61]. Exercise is associated with increased plasticity and neuroprotection in the region [61,62]. Interpretation of the inhibition of the region seen in the current studies exercised group would be facilitated by an investigation into the effect on connectivity and behavior to determine if exercise helps or harms susceptibility to cocaine addiction. A hypothesized brain circuit of regions found to have modified BGluM is pictured in Figure 3; however, further investigation is critical to validating or correcting this hypothesized circuit. This study cannot concretely identify functional connectivity.

The activation of the basal lateral amygdala observed in the males of the current experiment and nearby regions may have some implications for addiction behavior. The amygdala has been found to be involved in responses to drug cues and the reinstatement of cue-induced drug seeking [63,64]. The activation of the region is associated with relapse to drug-seeking, particularly by way of glutamatergic transmission [65,66]. Drug use has been found to decrease glutamate levels in the brain while sensitizing glutamatergic response to drug administration [67]. While the activation observed in the current study may imply increased susceptibility to relapse following exercise, the mechanism of action by which activation occurs hinders drawing concrete conclusions. In addition to increased activation, altered connectivity has been observed in the amygdala following cocaine abuse [68]. Dysfunction in connections with the prelimbic cortex has been implicated in cocaine reinstatement [69,70]. Exercise has been shown to induce plasticity [71,72] in the amygdala, which may help correct dysfunction; however, further research is necessary. Cocaine has also been shown to dysregulate glutamate signaling in the amygdala [67]. Glutamate agonism has been shown to help reduce the rewarding effects of cocaine [73,74] and enhance the extinction of cocaine CPP [75]; however, agonism has also been shown to enhance the reconsolidation of cocaine-associated memories [76]. Exercise has been shown to enhance glutaminergic transmission in the amygdala [77], which could have helpful or harmful effects on cocaine addiction. Behavioral evidence shows that exercise decreases relapse potential [77], but the role of the amygdala, its connections, and glutamatergic transmission require further investigation.

The observed activation of V2L may have a beneficial impact on cocaine abuse. In the visual cortex, cocaine has been found to reduce activity [78], gray matter [79], and 123 I uptake, indicating impaired function [80]. However, in response to cocaine cues, increased activation has been observed in comparison to food cues, indicating involvement in drug conditioning by facilitating the establishment of cocaine–stimulus associations [81,82]. Increased sensitivity to drug cues in this cortex has been observed for a wide array of drugs [83] in addition to cocaine [81,82], contributing to compulsive drug use. In the current study, exercise helped to ameliorate inactivation of the visual cortex by cocaine. The activation of the visual cortex via exercise may be able to disrupt drug cue conditioning and affect relapse susceptibility due to its ability to influence visual cortex plasticity [84,85]; however, further behavioral research is necessary.

The piriform cortex was observed to be significantly more activated in the exercised group. In the piriform cortex, acute cocaine use has been found to decrease [1–14C]octanoate labeling, which is associated with decreased function [86], in addition to reducing both dopamine and 5-HT synthesis [87]. Similarly, withdrawal (at 6 h and 72 h from cessation) [85] from the self-administration of cocaine (≥7 days for 3 h, followed by 12 h binge before) has been found to reduce regional cerebral metabolic rate for glucose [88] in the piriform cortex, which is associated with decreased function [88]. Exercise appears to impact this region in humans, as it has been observed that following exercise, regional cerebral blood flow in the region is decreased [89]. Connectivity between the piriform cortex and other regions is associated with cue-induced drug reinstatement and self-administration. For example, disconnection of projections between the piriform cortex and the orbitofrontal cortex has been found to reduce cue-induced fentanyl restatement [90]. Connectivity to the lateral habenula has been found to be differentially involved in compulsive methamphetamine-taking based on rats’ sensitivity to punishment [91]. Compulsive drug-taking was assessed using methamphetamine self-administration along with concomitant footshock. Connectivity between the piriform cortex and lateral habenula was positively associated with compulsive intake in punishment-resistant rats; the opposite was found in punishment-sensitive rats. Based purely on BGluM, it would appear that exercise would help attenuate cocaine’s effects on the piriform cortex; however, its impact on connectivity to regions, such as the lateral habenula and orbitofrontal cortex, should also be considered and investigated.

The observed activation of the trigeminothalamic tract of the exercised group may potentially influence the sensitivity of rats and thereby affect compulsive drug intake via the mechanism proposed for the piriform cortex. The trigeminothalamic tract is involved in orofacial nociception and motor innervation in the jaw [57,58]. The activation of this region suggests enhanced pain signaling, which would imply that the exercised group is more akin to the punishment-sensitive group seen in [91] in relation to orofacial pain. Past research following the same chronic exercise regimen as the current paper has linked acute cocaine administration with insular cortex activation [7]. In particular, chronic aerobic exercise and acute cocaine was found to increase BGluM in the granular and dysgranular regions of the insular cortex. Granular activation is also involved in orofacial proprioception and motor activation of the jaw [92]. The similarity between the trigeminothalamic tract and the granular region of the insular cortex raises the question of whether their involvement in cocaine-induced modification of orofacial nociception is related. The involvement of both in jaw movement may be related to cocaine-related jaw tension and vasoconstriction, as previously suspected [7]; however, more research is necessary.

In the exercised group, increased BGluM was observed in the perirhinal cortex. Cocaine withdrawal is associated with increased activity in the perirhinal cortex as well as increased c-fos expression [93]. Acute cocaine use has been shown to increase dopamine and 5-HT [94]. Cocaine has been found to enhance memory consolidation by D2R agonism, which may contribute to cocaine conditioning [95]. Cocaine also induces tPA mRNA in the perirhinal cortex. tPA is related to plasticity and may be involved in behavioral changes observed with cocaine use [96]. This information on the effects of cocaine on the perirhinal on its face would appear to show that exercise would have an adverse effect on drug abuse. However, research suggests that the effect of activation may be more complex and not simply additive. Chemogenetic activation of the perirhinal cortex has been found to reverse methamphetamine-induced NOR task impairment as well as reduce relapse [97]. Similarly, mGlu5R activation in the perirhinal cortex reduces methamphetamine relapse while recovering NOR test performance [98]. Exercise appears to influence the perirhinal cortex in a similar manner, as evidenced by observed improvements in NOR tasks [99,100]. Corticotropin-releasing factor 2 receptors have been implicated in NOR task deficits seen during cocaine withdrawal [93]. Exercise is known to impact the corticotropin system and could potentially counteract the effects of cocaine via this mechanism. This evidence supports further investigation into the ability of exercise to ameliorate perirhinal cortex-mediated cocaine relapse and NOR deficits akin to that observed in methamphetamine via perirhinal activation.

A meta-analysis of the effectiveness of short-term exercise on drug rehabilitation found improvements in drug craving, cognitive functioning, and perceived stress among exercised individuals [101]. This indicates that animal models investigating the effects of exercise on drug addiction have some translatability into humans. The current study provides anatomical targets for further investigation into the underlying mechanisms involved in the observed improvements in drug rehabilitation. An important note is that for cocaine specifically, the use of exercise as an intervention for cocaine intake has mixed results in humans. Treadmill running has been found to non-significantly improve cocaine abstinence and reduce craving [102]. Non-significance could be attributed to methodological limitations such as the small sample size; however, mechanisms by which exercise may enhance cocaine response, such as the activation of the amygdala and perirhinal cortex, should also be considered. Identifying potential mechanisms by which exercise may enhance cocaine response can help develop more specific interventions. These may include altered type and intensity of exercise or coadministration of pharmacological agents to minimize and/or inhibit mechanisms by which exercise could exacerbate cocaine response. A higher-powered study demonstrated the beneficial effect of exercise on stimulant rehabilitation [103], though benefits are reduced among black participants who are known to use cocaine at higher rates. While many other factors may be involved, the limitation of exercise as an intervention for cocaine use specifically must be considered. While it is critical to identify limitations, it is also important to note that the literature overall demonstrates a beneficial effect of exercise in reducing cocaine response in humans [101,102,103,104,105].

## 5. Conclusions

Treatments, even adjuncts to treatment, are needed for patients with cocaine use disorders. Exercise, such as in vigorous physical regimens, rather than sitting and going to meetings, has been discussed in recovery forums and by celebrities who have used this method to recover. Eminem credited exercise with his recovery from cocaine and other drug addictions [106]. The dose, duration necessary, how to monitor fidelity to the protocol and brain plasticity are all questions we need to ask in laboratory models and translate to humans.

The results demonstrated that compared to sedentary controls, chronically exercised rats were observed to have modulated brain glucose metabolism in seven regions. The SIBF primary somatosensory cortex was found to be inhibited in the exercised group. Increased brain glucose metabolism was observed in the amygdalopiriform transition area, piriform cortex layer 1, trigeminothalamic tract, perirhinal cortex rhinal fissure, basolateral amygdaloid nucleus dorsal endopiriform nucleus, and secondary visual cortex, lateral area. The literature shows that the regions modulated are involved in cocaine response. However, further research is needed to elucidate changes in behavior and regional connectivity, especially in humans. 

## Figures and Tables

**Table 1:** Brain regions with significant decreases at *p* < 0.001 and voxel threshold K > 50 in BGluM between exercised and sedentary groups following chronic exercise and chronic cocaine exposure are labeled as inhibited. Regions with significant increases (*p* < 0.001, K > 50) in BGluM are labeled as activated. Increases are interpreted as activation while inhibitions are interpreted as deactivation. Cluster location is indicated by coordinates in stereotaxic space (medial-lateral, anterior–posterior, and dorsal–ventral). The t-values and z-scores were calculated from the average BGluM of all voxels within the significant clusters. KE represents the number of voxels in the respective cluster. Each cell under “Brain Region(s)” represents a separate cluster.

Brain Region (s)	Activated or Inhibited	ML (mm)	DV (mm)	AP (mm)	t Value	z-Score	KE
Primary somatosensory cortex (SIBF)	Inhibited	44	26	−44	4.85	3.32	99
Piriform cortex (Pir)	Activated	−58	104	−28	6.53	3.87	94
Piriform cortex	Activated	−58	104	−28	6.53	3.87	94
Amygdalopiriform transition (Apir)	Activated	56	100	−42	6.39	3.83	244
Trigeminothalamic tract (TTH)	Activated	10	84	−60	5.57	3.58	178
Basolateral amygdaloid nucleus, dorsal (BLA/DEN)	Activated	52	90	−20	4.56	3.2	67

## Data Availability

The data presented in this study are available in the article.
